# Involvement of *Cis*-Acting Elements in Molecular Regulation of JH-Mediated Vitellogenin Gene 2 of Female *Periplaneta americana*

**DOI:** 10.3389/fphys.2021.723072

**Published:** 2021-08-30

**Authors:** Azza M. Elgendy, Amr A. Mohamed, Bernard Duvic, Muhammad Tufail, Makio Takeda

**Affiliations:** ^1^Department of Entomology, Faculty of Science, Cairo University, Giza, Egypt; ^2^Department of Agrobioscience, Graduate School of Agricultural Science, Kobe University, Hyogo, Japan; ^3^DGIMI, Univ Montpellier, INRAE, Montpellier, France; ^4^Ghazi University, Dera Ghazi Khan, Punjab, Pakistan

**Keywords:** vitellogenesis, juvenile hormone, 20-hydroxyecdysone, *cis*-regulatory elements, hemimetabola, *periplaneta*, transcriptional regulation

## Abstract

Vitellogenins (Vgs) are yolk protein precursors that are regulated by juvenile hormone (JH) and/or 20-hydroxyecdysone (20E) in insects. JH acts as the principal gonadotropin that stimulates vitellogenesis in hemimetabolous insects. In this study, we cloned and characterized the *Periplaneta americana Vitellogenin 2* (*Vg2*) promoter. Multiple sites for putative transcription factor binding were predicted for the 1,804 bp *Vg2* promoter region, such as the Broad-Complex, ecdysone response element (EcRE), GATA, Hairy, JH response element (JHRE), and Methoprene (Met)-binding motif, among others. Luciferase reporter assay has identified that construct −177 bp is enough to support JH III induction but not 20E suppression. This 38 bp region (from −177 to −139 bp) contains two conserved response element half-sites separated by 2 nucleotides spacer (DR2) and is designated as *Vg2*RE (^−168^GAGTCACGGAGTCGCCGCTG^−149^). Mutation assay and luciferase assay data using mutated constructs verified the crucial role of G residues in *Vg2*RE for binding the isolated fat body nuclear protein. In *Sf*9 cells, a luciferase reporter placed under the control of a minimal promoter containing *Vg2*RE was induced by JH III in a dose- and time-dependent manner. Nuclear proteins isolated from previtellogenic female fat body cells bound to *Vg2*RE, and this binding was outcompeted by a 50-fold excess of cold *Drosophila melanogaster* DR4 and *Galleria mellonella* JH binding protein response elements (Chorion factor-I/Ultraspiracle). Affinity pull-down experiment with nuclear extracts of previtellogenic female fat body, using 31-bp probe *Vg2*RE as bait, yielded a 71 kDa candidate nuclear protein that may mediate the regulatory action of the JH III.

## Introduction

The American cockroach, *Periplaneta americana*, thrives in temperate climate bands across the globe. Cockroaches live in houses, groceries, stores, and hospitals causing disease in humans, such as allergic reactions that include lung and skin reactions. They are also potential mechanical vectors for several pathogens, such as *Clostridium perfringens, Pseudomonas aeruginosa*, and *Salmonella enterica* serotype Bredeney causing anthrax, cholera, and diphtheria, respectively (Baumholtz et al., 1997; Atiokeng Tatang et al., 2017). Their widespread occurrence is due to a high reproductive capacity and rich nutrients supplement to the future developed embryos by female cockroaches. Hence, the synthesis of vitellogenin (Vg), the major yolk protein precursors, in the fat body culminates the female reproductive success, and the underlying mechanisms have attracted the substantial interest of researchers. However, so far, few studies have investigated the hormone-mediated transcriptional regulation of Vg in hemimetabolous insects (Elgendy et al., 2019; Wu et al., 2021).

Juvenile hormone (JH), the insect-specific sesquiterpenoid hormone, and the molting hormone, 20-hydroxyecdysone (20E), are the two major hormones that govern the reproduction of female insects (Liu et al., 2018; Li et al., 2019). JH is the dominant controlling hormone in most hemimetabolous insects, such as Orthoptera (*Locusta migratoria*) (Glinka and Wyatt, 1996; Song et al., 2014; Luo et al., 2017), Blattodea (*Blattella germanica, Diploptera punctate*, and *P. americana*) (Comas et al., 1999, 2001; Marchal et al., 2014; Kamruzzaman et al., 2020), and Hemiptera (*Nilaparvata lugens, Pyrrhocoris apterus*, and *Cimex lectularius*) (Smykal et al., 2014; Gujar and Palli, 2016; Lu et al., 2016a,b). In holometabolous insects, such as *Tribolium castaneum*, although 20E is involved in reproduction, JH is still the primary hormone governing reproduction (Parthasarathy et al., 2010). Vitellogenesis in many lepidopteran species occurs prior to adult emergence, where 20E dominates (Ramaswamy et al., 1997). The 20E is the leading regulator in dipterans, such as mosquitoes and flies, but JH still plays a key role in 20E-mediated events (Raikhel et al., 2005).

Extensive studies have indicated the involvement of the JH receptor Methoprene-tolerant (*Met*) in the antimetamorphic JH signaling. Met has a conserved role in JH-controlled processes in both hemimetabolous and holometabolous insects, such as oogenesis, oviposition, and regulation of Vg mRNA levels, fecundity reduction, mating regulation, sex pheromone production, and regulation of lipid metabolism (Minakuchi et al., 2008, 2009; Parthasarathy et al., 2008, 2010; Konopova et al., 2011; Lozano and Bellés, 2011; Kayukawa et al., 2012; Zou et al., 2013; Marchal et al., 2014). In contrast, there are very few reports on the role of Met, and other JH binding proteins, such as Hairy, CF-II (Chorion factor 2), FoxO (fork head transcription factor), and Br-CZ (Broad-Complex) during reproductive events in the insects (Mao et al., 2020; Tsang et al., 2020). For example, Li et al. (2018) have determined that insulin/IGF signaling (IIS) and target of rapamycin (TOR) indirectly promote *P. americana* vitellogenesis through JH-Met/Kr-h1 Krüppel homolog 1 that form a positive feedback regulatory loop *via* the doublesex (Dsx).

In many adult insects, such as *T. castaneum, L. migratoria, Helicoverpa armigera, P. apterus, N. lugens, Bactrocera dorsalis*, and *Sogatella furcifera*, JH requires the Met/Taiman (Tai) complex to achieve its previtellogenic and vitellogenic regulations on the fat body competency and Vg synthesis (Parthasarathy et al., 2010; Zou et al., 2013; Guo et al., 2014; Smykal et al., 2014; Song et al., 2014; Lin et al., 2015; Gujar and Palli, 2016; Wang et al., 2017; Ma et al., 2018; Yue et al., 2018; Hu et al., 2019). *L. migratoria*, with a panoistic ovary where terminal oocytes are ovulated together, has been a longstanding model system for studying JH-dependent female reproduction (Wyatt and Davey, 1996; Roy et al., 2018). Detection of Met/Tai as the nuclear JH receptor helps to understand the regulation of JH-induced transcription, controlling insect metamorphosis, and reproduction (Gijbels et al., 2019).

The molecular mechanisms of JH regulation in insect vitellogenesis and oogenesis are still obscure. The fact that *Vg* is directed by an endogenous *cis*-acting promoter has been investigated in *Aedes aegypti* and *Anopheles stephensi* through a transgenic approach (Nirmala et al., 2006; Kokoza and Raikhel, 2011). However, how the *Vg cis*-regulatory elements regulate gene expression in hemimetabolous insects such as *P. americana* is still unclear. Elucidating the *Vg cis*-regulatory elements in *P. americana* may, thus, contribute to further understanding the physiological roles of Vg and the advancement of genetic manipulation technologies, e.g., RNA interference (RNAi)-based population management *via* RNAi-aided knock-down of Vg isoforms and their regulatory gene(s), suppressing egg formation and embryonic development of this pest.

In the previous studies, in *P. americana*, we identified a direct repeat separated by a 2-nucleotide spacer designated, *Vg1*HRE, that is similar to the *Drosophila* ecdysone response element (EcRE) direct repeat 4 (DR4) (Elgendy et al., 2019). Moreover, nuclear proteins were successfully bound to *Vg1*HRE which suggested that the *P. americana Vg1* (*PaVg1*) is hormonally regulated by transcription factors interacting with the upstream regulatory elements found in the *Vg1* promoter region, such as other insect *Vg* genes (Chen et al., 2004; Zhu et al., 2007; Lin et al., 2017; Elgendy et al., 2019).

The present study aimed to clarify the molecular regulatory mechanism of *Periplaneta americana vitellogenin* gene 2 (*Vg2*) by JH III. A cloned 1,804 bp promoter region of *Vg2* from *P*. *americana* is characterized by several putative *cis*-regulatory elements. The luciferase reporter system identified a 204 bp promoter region that will support JH III induction and 20E suppression. This promoter region harbors a *cis*-acting element named *Vg2*RE. The non-vitellogenic female nuclear protein extract (NPE) contains a 71 kDa protein that specifically binds to the newly identified *Vg2 cis*-acting elements, *Vg2*RE. This candidate nuclear protein is still under study. These findings suggest that *Vg2*RE efficiently drives the expression of *Vg2* to support sufficient yolk formation for the developed embryo through the classical JH molecular action including nuclear protein candidates, such as Met receptor and FoxO protein. However, other identified *cis*-binding elements imply the complexity of such regulation and the induction and/or repression of *Vg2* expression during repetitive vitellogenic cycles.

## Materials and Methods

### Insects

Colonies of *P. americana* were maintained as described in Elgendy et al. (2019). Fat bodies from nymphs (last instar) and adult males and females were collected for the preparation of nuclear extracts and gDNA isolation.

### Promoter Sequencing and Primer Extension Analysis

The *Vg2* promoter has been cloned as described by Elgendy et al. (2019) with the primers *Vg2*-Prom1 and *Vg2*-Prom2 (Supplementary Table 1). The longest promoter fragment was subcloned into the pGL3-Basic vector (Promega, Madison, WI, USA). The transcription start site (TSS) was experimentally determined using a primer extension kit (Promega) and γ-^32^P ATP end-labeled reverse primer (*Vg2*p-R) following the instruction manual.

Fat bodies were collected from 1-day-old females. Genomic DNA was isolated using the Mammalian Genomic DNA Kit (Sigma, St. Louis, MO, USA). The purity of the nucleic acid samples was assessed based on the A260/A280 ratio (Sambrook and Russell, 2001). The *Vg2* promoter sequence was identified using the BD GenomicWalker™ Kit (BD Bioscience, Clontech, San Jose, CA USA). For this purpose, six genomic DNA libraries were constructed by digesting genomic DNA with DraI, EcoRV, HincIII, PuvI, SspI, and StuI. The resulting DNA fragments were purified and ligated to GenomeWalker adaptors. Further, 1 μl of each DNA library was subjected to primary PCR with AP1 (provided with the kit) and *Vg2*-Prom1 primer (Supplementary Table 1). The primary PCR mixtures were diluted and used as templates for secondary PCR with AP2 (provided with the kit) and *Vg2*-Prom2 primer. Secondary PCR products were inserted into pT7Blue, a TA cloning vector (Novagen, Nottingham, UK). Sequencing was performed using the ABI Prism BigDye Terminator Cycle Sequencing Kit (PE Applied Biosystems, Foster, CA, USA) and a 3100 Genetic Analyzer sequencer (PE Applied Biosystems). The longest promoter fragment was subcloned into the pGL3-Basic vector (Promega, Madison, WI, USA). The TSS was experimentally determined using a primer extension kit (Promega) and γ-^32^P ATP end-labeled reverse primer (*Vg2*p-R) following the instruction manual.

### Plasmid Construction

The 1,804-bp 5′ flanking region of the *PaVg2* promoter was cloned between the SacI and HindIII (Takara, Kyoto, Japan) sites of the pGL3-Basic vector. Luciferase reporter constructs with different length deletions of the *PaVg2* promoter were cloned using sense primers with Sacl at their 5′ ends and an antisense GL-2 primer (Supplementary Table 1). The sequences of the constructs were confirmed using the GL-2 and RV3 primers. The pGL3-Basic vector was used as an internal control for transfection efficiency.

### Site-Directed Mutagenesis

Site-directed mutagenesis was carried out on the *in silico* identified putative *Vg2*RE from −169 to −139 bp. The base change was done using the site-directed, ligase-independent mutagenesis (SLIM) methodology as described by Chiu et al. (2008) and primers sequence in Supplementary Table 2. An aliquot of 20 μl from each reaction was used for transformation. The clones were screened by sequencing for the desired mutations.

### Cell Culture, Transfection, and Luciferase Assay

*Spodoptera frugiperda Sf*9 cells were cultured in SF900 II SFM supplemented with 10% fetal bovine serum (Life Technologies Polska, Poland) and 50 μg/ml gentamycin (Sigma) at 28°C. Transient transfections of *Sf*9 cells were performed as described previously by Elgendy et al. (2019). Briefly, *Sf*9 cells were seeded in a 12-wells plate (6 × 10^5^ cells/well) that was incubated overnight at 28°C before transfection. Further, 1 μg of each reporter construct was co-transfected with 0.5 μg of pRL-Actin5C using 4 μl of Tfx™-20 reagent (Promega) in 400 μl of serum-free media (SFM; GIBCO, Invitrogen, MA, USA) for 12 h. Then, the transfection mixture was removed, and 1 ml of growth medium (SF900 medium with 10% fetal bovine serum) was added to each well. The growth medium was added and after 48 h the cells were harvested and lysed using 100 μl of lysis buffer (Promega). The cells were frozen at −70°C, thawed to enhance cell lysis, centrifuged at 4°C for 2 min, and 20 μl of the supernatant was used for the assay. The assay was performed using the Bright-Glo™ Luciferase Assay System (Promega) and an ATTO Luminescencer PSN (ATTO, Tokyo, Japan). The results were normalized against *Renilla* luciferase activity and plotted as relative luciferase activity (RLA); each experiment was repeated three times.

To study the time-course of the hormonal effect, *Sf*9 cells were transfected with 1 μg of each reporter construct and 3 μl of Tfx™-20 reagent (Promega) in 400 μl of SFM (GIBCO, Invitrogen) for 12 h, at which time the transfection mixture was removed and 1 ml of growth medium (SF900 medium with 10% fetal bovine serum) with or without hormone was added to each well. After 48 h, the growth medium was added, and cells were harvested and lysed with 200 μl of luciferase lysis reagent (Promega). Cells were frozen at −70°C and thawed to enhance cell lysis. The lysate was centrifuged at 4°C for 2 min and the supernatant was used for the assay. The assay was performed using the Bright-Glo™ Luciferase Assay System (Promega) and an ATTO Luminescencer PSN (ATTO, Tokyo, Japan). The results were normalized against total protein concentration; each experiment was repeated three times. Luciferase activity was normalized to the protein concentration per 20 μl of cell lysate supernatant used in each luciferase reaction.

### Preparation of Nuclear Extracts and DNA Binding Assay

Nuclear extracts were prepared from *Sf*9 and insect fat body cells according to methods described recently by Elgendy et al. (2019). To characterize the identified 31-bp, *PaVg2*RE, single-stranded oligonucleotides (Supplementary Table 1) were annealed to a double-stranded probe and end-labeled with γ-^32^P-ATP (MP Biomedical, Tokyo, Japan) by the DNA 5′-End-Labeling System (Promega). The oligonucleotides were then purified by passing the samples through MicroSpin G-25 Columns (GE Healthcare, IL, USA). In each binding reaction, 5–7 μg of nuclear protein and 0.1 pmol of the labeled probe were incubated in electrophoretic mobility shift assay (EMSA) buffer [25 mM HEPES pH 7.6, 5 mM MgCl_2_, 34 mM KCl, 50 ng/μl poly (dI-dC), and 10% glycerol] for 30 min at room temperature.

For phosphorylation, nuclear protein extract (NPE) was supplemented with adenosine triphosphate (ATP) to a final concentration of 1 mM and incubated at 22°C for 20 min before the binding reaction. For phosphatase treatment, NPE was incubated with 1.0 U calf intestinal alkaline phosphatase (CIAP; Takara) at 22°C for 20 min before adding the labeled probe. For the super-shift experiments, an antibody was added to the binding reaction, and the labeled probe was finally added. The bound DNA-protein complex was resolved using 5% non-denaturing polyacrylamide gels (prerun for 45 min) in 1 × Tris-borate-EDTA (TBE) buffer at a constant voltage of 150 V.

For the super-shift experiments, an antibody was added to the binding reaction, and the labeled probe was finally added. The bound DNA-protein complex was resolved using 5% non-denaturing polyacrylamide gels (prerun for 45 min) in 1 × TBE buffer at a constant voltage of 150 V. Super-shift experiments were performed using *Drosophila* monoclonal anti-EcR and anti-Ultraspiracle common region antibodies (AG 10.2; DDA 2.7). Antibodies were generously provided by Drs. C. Thummel (University of Utah, UT, USA) and C. Bass (EMBL Heidelberg, Germany). In addition, this study used polyclonal antibodies against *B. mori* EcR (AB) and USP (AB), which were generous gifts from Dr. H. Fujiwara (University of Tokyo, Japan). The gels were exposed to MP3 Hyperfilm (Amersham). The inhibitory region from −1,402 to −1,237 bps was amplified using PCR, purified from the agarose gel, and then labeled and used for EMSA as previously described.

Many response element oligonucleotides *Vg1*RE hspDR4, hspIR, jhbp21, JHRE, and CF1/USP (Supplementary Table 1) were purchased from Invitrogen (Tokyo, Japan). For DNA binding studies, a pair of sense and antisense oligonucleotides were annealed, and the quality and concentrations of the oligonucleotides were verified by polyacrylamide gel electrophoresis (PAGE) before use in the competition assays. A maximum of 50-fold more oligonucleotide than the labeled *Vg2*RE probe was used in each reaction. The binding reaction was performed as described for EMSA, and the competitor oligonucleotide was added simultaneously with the labeled *Vg2*RE probe.

### Pull-Down Assay

The 31 bp (*Vg2*RE) wild-type forward and reverse probes were synthesized, biotinylated at the 5′ end of the sense strand, and annealed in equimolar amounts. Then, Dynabeads M-280 streptavidin (Dynal, Inc., Lake Success, NY, USA) were washed three times in phosphate-buffered saline (pH 7.4) containing 0.1% BSA and two times with Tris-EDTA containing 1 M NaCl. Between washes, the beads were pulled down by a magnet (Promega). Each double-stranded biotinylated DNA probe (10 pmol) was assayed with 250 μg of beads, and the mixture was incubated for 40 min at room temperature with continuous mixing. The beads were then washed two times in Tris-EDTA with 1 M NaCl and two times in 1 × binding buffer (EMSA binding buffer). Fat body nuclear extracts (35 μg) were preincubated for 5 min in 1 × binding buffer with 2.0 μg of poly (dI-dC) in a total volume of 100 μl. Dynabeads M-280 streptavidin (250 μg) bound to the probe (10 pmol) were then added to the mixture and incubated for an additional 20 min at room temperature. Proteins bound to the probe were pulled down with a magnet, and then, the beads were washed once with × 0.5 binding buffer containing 0.5 μg/ml and subsequently pulled down. The beads were re-suspended in × 1 sample buffer and boiled for 10 min. Then, the beads were subjected to 10% sodium dodecyl sulfate–polyacrylamide gel electrophoresis (SDS-PAGE). Electrophoresis was performed at 200 V for 50 min, and protein bands were visualized by silver staining (Silver Stain Plus, Bio-Rad, CA, USA).

### Statistical Analysis

The experiments were conducted with three independent replications (transfections at three different times), and on each occasion were performed three times. An unpaired Student's *t*-test was used for comparison of the two data sets. In all cases, *P* < 0.05 were considered significant. Statistical analysis was performed with IBM-SPSS Statistics v.22 (IBM, Armonk, NY, USA).

## Results

### Sequencing of *Vg2* Promoter and Search for Potential Regulatory Elements

**Figure 1:**
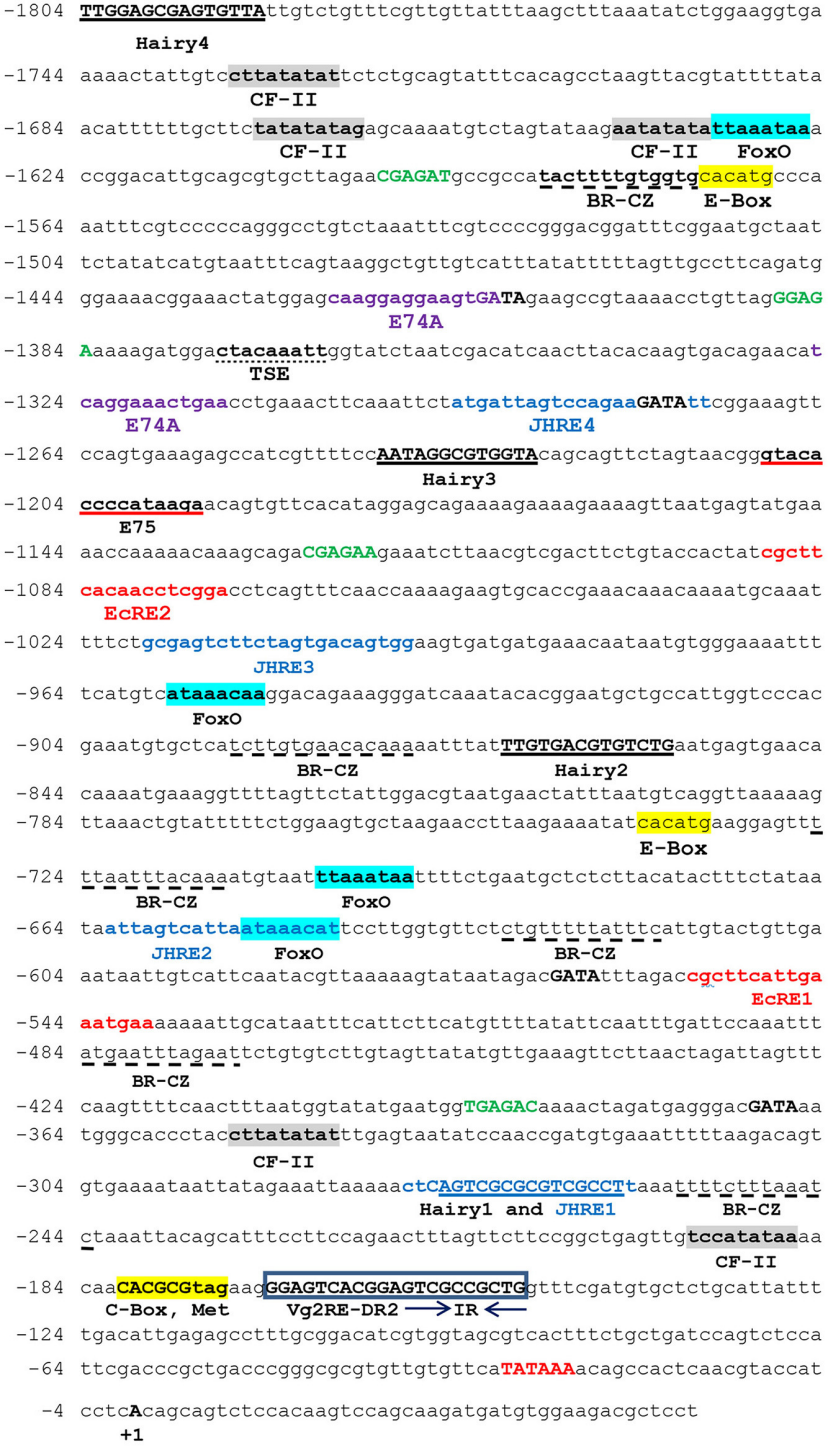
Nucleotide sequence of the *Vitellogenin 2* (*Vg2*) promoter. The nucleotides are numbered from the transcription start point as +1. The name of the binding factor is written under its consensus sequence. TATA box, GAGA, and GATA elements are shown in bold red, bold green, and bold black capital letters, respectively. Juvenile hormone response element (JHRE): blue color. Ecdysone response element (EcRE): red color. Broad complex (Br-CZ) dashed underline. Chorion factor (CF-II): gray box. Hairy is underlined. E-Box and C-Box: yellow box. E74A: violet color. E75: red underline. FoxO: blue box. Tissue-specific expression (TSE): dot underlined. The direct repeat *Vg2*RE-DR2 is bold, inside frames. Arrows indicate the directions of the inverted repeats IR.

**Figure 2:**
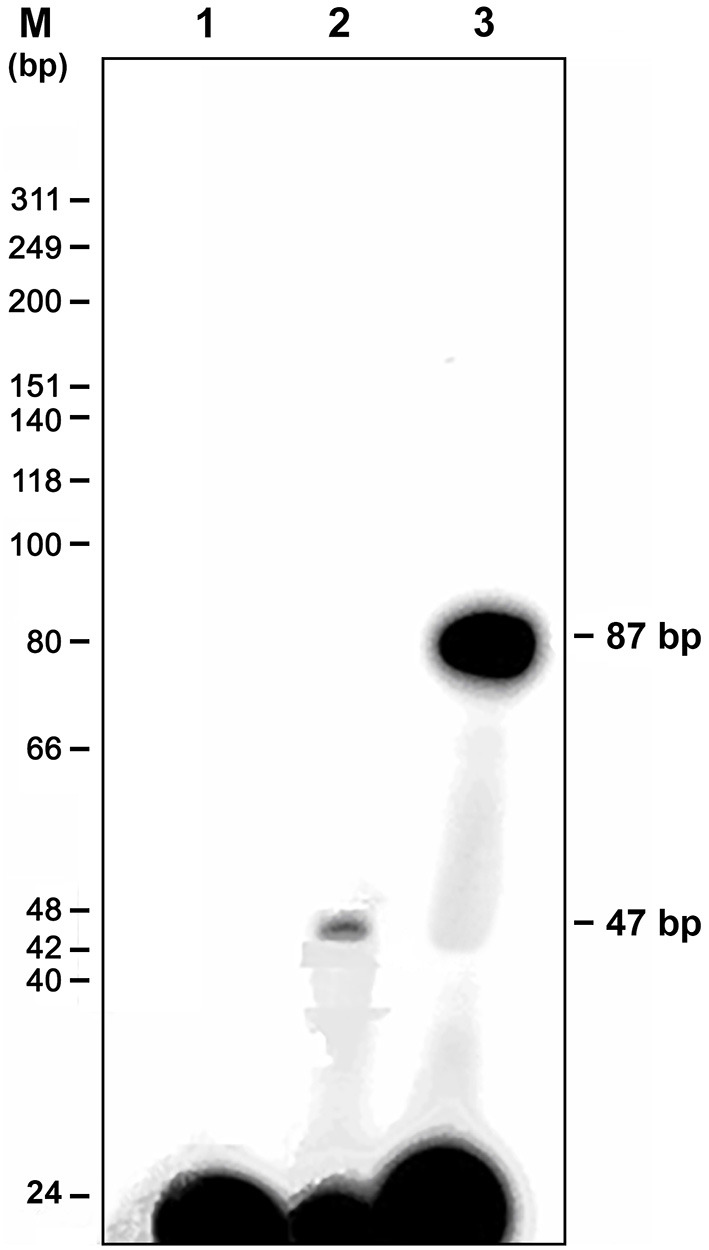
Mapping of the transcription start site (TSS) by primer extension analysis. A primer extension kit (Promega) was used in accordance with the instructions from the manufacturer; mRNA was prepared from a 4-day-old female fat body. A 22-bp synthetic oligonucleotide complementary to nt 18–39 of *Vg2* was synthesized, end-labeled with ^32^P ATP, and used as a primer to produce a single product that was 47 bp in length (lane 2). The DNA marker and a negative control without mRNA are shown in lanes M and 1, respectively. The 87 bp product is the positive DNA control supplied with the kit (lane 3).

The genomic walking procedure was used to clone and sequence the *Vg2* promoter of *P. americana* (*PaVg2*). A 1,850 bp genomic fragment was cloned into the TA cloning vector pT7blue (Novagen), sequenced from both directions (Figure 1), and deposited in GenBank (AB449028). This genomic sequence includes a promoter region from −1 to −1,804 and cDNA fragment from +1 to +46 including 11 nucleotides extension from the previously cloned *Vg2* cDNA by Tufail et al. (2001). The genomic region from +46 to −1,804 showed a typical TATA box at −30 bp upstream of the identified TSS (Figure 1). The TSS was determined using the TRANSFAC program and confirmed by primer extension assay (Figure 2) and numbered as +1.

The *Vg2* promoter (+46 to −1,804 bp) was examined for potential regulatory elements with Match and Patch 2.0 software, available at BIBASE (http://www.generegulation.com/wolfenbuttel). The region was also compared by visual inspection to the structures of many other insect gene promoters. The analysis showed a match with half-sites of several known response elements, such as TATA box-binding protein, EcRE, CF-II, E74A, FoxO, Hairy, GATA, GAGA, and E-box repeat, and the cAMP response element (CRE) (Figure 1; Table 1). In addition to tissue-specific expression elements, TSE (CTACAAAGT) from *Drosophila* yolk protein gene promoter (Abrahamsen et al., 1993), CF/USP ${(}^{\text{A}}{/}_{\text{G}}^{\text{G}}{/}_{\text{T}}\text{GGTCA)}$ elements from *jhbp* promoter of *G. mellonella* (Sok et al., 2008), JH response elements (GAGGTTCGAG^A^/_T_ CCT^T^/_C_) from *jhp21* gene of *L. migratoria* (Zhou et al., 2002), and (AGATTA) from *JH esterase* gene promoter of spruce budworm *Choristoneura fumiferana* (Kethidi et al., 2004) were also identified in *PaVg2* promoter.

**Table 1:** Possible regulatory elements located in the promoter region of the *Vg2* gene.

**Regulatory element**	**Sequences**	**Position**	**Homology**
JHRE	ATGATTAGTCCAGAAGATATT	−1,294	Budworm *JHE*
	GCGAGTCTTCTAGTGACAGTGG	−1,019	*L*. **migratoria* Vg*
	ATTAGTCATTAATAAACA	−662	*G. *mellonella** JHBP
	CTCAGTCGCGCGTCGCCT	−278	
EcRE	CGCTTCACAACCTCGGA	−1,089	*Drosophila* 20E-responsive gene
	CGCTTCATTGAAATGAA	−555	
E-box	CACATG	−1,574	
	CACATG	−739	
C-box	CACGCG	−181	
Met	CACGCGTAG	−181	
CRE	ATTTAT	−1,471 and −488	
	ATGAAT	−484 and −401	
	CATTGT	−618	
E74A binding site	CAAGGAGGAAGTGA	−1,424	
	TCAGGAAACT	−1,325	
E75 binding site	GTACACCCCATAAGA	−1,209	
GATA binding site	TGATAG	−1,413	
	AGATAT	−1,280	
	CGATAT	−567	
	CGATAA	−371	
GAGA binding site	CGAGAT	−1,600	
	GGAGA	−1,388	
	CGAGAA	−1,126	
	TGAGAC	−393	
TSE element	CTACAAATT	−1,373	
RXR binding site	ACTGAACCTG	−1,318	Mammalian retinoid-X receptor
	GAAGTGCACC	−1,054	
FoxO	TTAAATAA	−1,633	
	ATAAACAA	−957	
	TTAAATAA	−705	
	ATAAACAT	−651	
Br-CZ	TACTTTTGTGGTG	−1,587	
	TCTTGTGAACACAA	−892	
	TTTAATTTACAAA	−725	
	CTGTTTTTATTTC	−630	
	ATGAATTTAGAAT	−484	
	TTTTCTTTAAAT	−256	
Hairy	TTGGAGCGAGTGTTA	−1,804	
	AATAGGCGTGGTA	−1,240	
	TTGTGACGTGTCTG	−870	
	AGTCGCGCGTCGCCT	−275	
CFII	CTTATATAT	−1,732	
	TATATATAG	−1,670	
	AATATATAT	−1,641	
	CTTATATAT	−352	
	TCCATATAA	−195	
Vg2RE-DR2	GGAGTCACGGAGTCGCCGCTG	−169	

### Deletion Analysis of the *Vg2* Promoter and Identification of Juvenile Hormone Response Element Region

**Figure 3:**
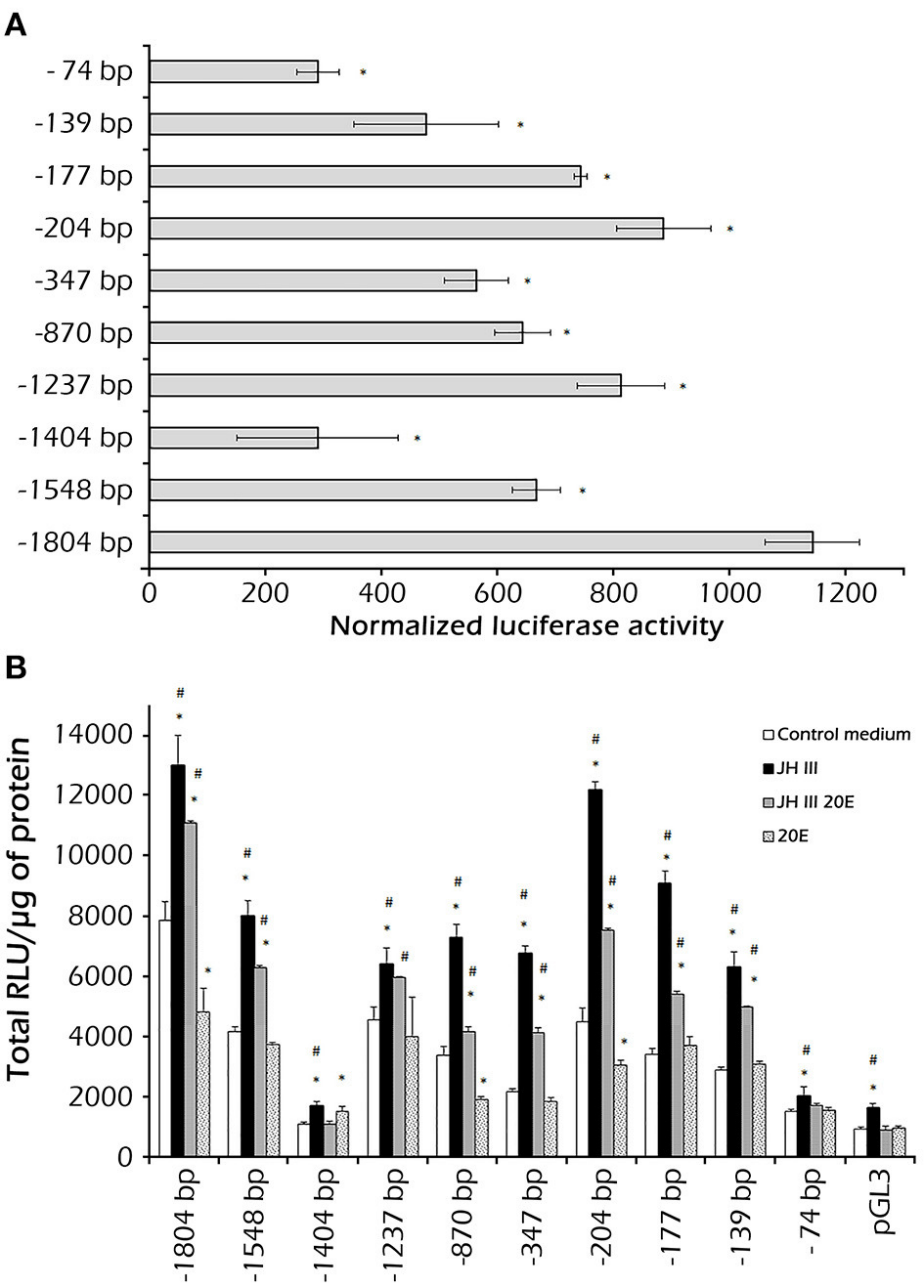
Identification of the juvenile hormone (JH) responsive region in the *Vg2* promoter. A 1,804-bp DNA fragment upstream of the TSS was isolated, and 5′ deletion fragments (−1,548, −1,404, −1,237, −870, −347, −204, −177, −139, and −74 bp) were produced by PCR and cloned into a luciferase reporter vector (pGL3). These constructs were assayed in *Sf*9 cells. In this, 1 μg of each reporter plasmid construct was transfected with 3 μl of Tfx™ −20 reagent. After 12 h, the cells were treated with medium alone or medium with 1 μM JH III, 1 μM 20E, or 1 μM JH III, and 1 μl 20E. Luciferase activity was assayed 48 h after treatment. **(A)** The assay was performed using the Bright-Glo™ Luciferase Assay System (Promega) and results were normalized against *Renilla* luciferase activity and plotted as relative luciferase activity (RLA); each experiment was repeated three times. Values are presented as the mean ± SD. **(B)** For each reporter plasmid construct, luciferase activity was assayed 48 h after the addition of hormones JH III, 20E, and both JH III and 20E mixture. Data are presented as mean ± SD. Bars marked with ^*^ or # are significantly different (*P* < 0.05) as compared to the media (control media without hormones) or the combined JH III and 20E treatment, respectively.

Functional tests in the *Sf*9 cell line showed that the constructs containing various lengths of fragments of the *PaVg2* promoter resulted in different transcriptional activity. As might have been expected, the construct containing the core promoter, with TATA box only (−74 bp), exhibited the activity almost equal to the empty vector pGL3-Basic (Figure 3B). Extending the promoter to position −139 caused an approximate one-third increase in the transcription activity in relation to the core promoter activity. This transcription activity stayed at this level, with some fluctuations, until the −1,404 bp construct, which showed a statistically significant decrease in the activity. This −1,404 bp construct contains an RXR response element, which is a homolog of CF/USP element II, at −1,318 bp and an E74E at −1,325 bp (Figure 1; Table 1). This decrease in transcription activity disappeared when the sequence was extended further, and the highest activity value was measured at −1,804 (Figure 3A). These results suggested that the 38-bp region between −177 and −139 is sufficient for a JH-induced response observed for the *PaVg2* gene (Figure 3B). This sequence contains direct repeat elements separated by a two-nucleotide spacer and shows 100% identity with consensus DR4 elements (Figure 1; Table 1). This 38-bp region is designated as a putative JHRE (*Vg2*RE).

### The Effect of Hormones on the Transcription Activity of the *PaVg2* Promoter in *Sf*9 Cell Line

**Figure 4:**
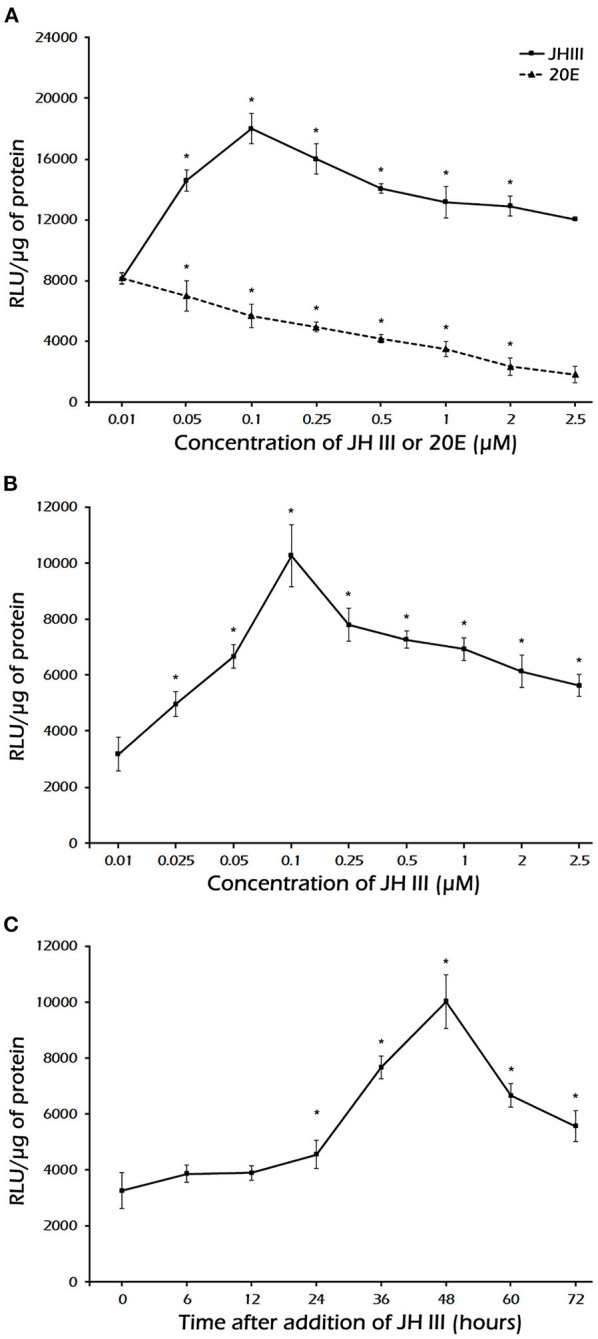
**(A)** Dose-response induction by JH III and inhibition by 20E of luciferase expression under the control of the *Vg2* full-length promoter. **(B)** Dose-response induction of luciferase expression under the minimal promoter length −204 bp. **(C)** Time course of JH III-mediated induction of luciferase expression under the control of a minimal promoter length −204 bp. The values are presented as the mean ± SD (*n* = 3). In these experiments, *Sf*9 cells were transfected with the pGL3 vector containing the *Vg2* promoter, cells were exposed to different concentrations of JH III or 20E, and luciferase activity was measured 48 h after hormone addition. ^*^Statistically significant at *P* < 0.05.

The JH III induction of reporter activity through the full length and −204 bp *PaVg2* promoter is both JH III dose- and exposure time-dependent (Figure 4). The induction of reporter activity started at as low as 25 nM JH III, augmented with increasing concentration of JH III, and peaked at 100 nM concentration of JH III (Figure 4A). The reporter activity decreased at JH III concentrations greater than 100 nM. This may be due to the potential cytotoxicity of *in vitro* hormone preparations to the cells. JH III itself is likely, not cytotoxic but is insoluble in an aqueous medium at a concentration higher than 10^−5^ M. Above that concentration, it can form a film on top of the medium that can disrupt the proper aeration of cell culture. The induction of reporter activity increased with time and reached a maximum at 48 h after adding the hormone. There was a decrease in reporter activity by 60 and 72 h after adding hormone (Figure 4C). Also, the minimum promoter length (−204 bp) showed the same induction pattern of the full-length promoter under exposure to a serial dilution of JH III (Figure 4B). The suppression of transcription activity was observed when 20E was added alone or mixed with JH III in both full-lengths (Figures 3B, 4A) and −204 bp of *Vg2* promoter (Figure 3B). The −1,404 bp and basic pLG3 constructs showed no effect upon hormone treatment, suggesting the presence of suppressor elements in the region between −1,404 and −1,237 bp. Control medium alone, without any hormones, had a limited effect on transcription activity.

### Characterization of *PaVg2*RE

**Figure 5:**
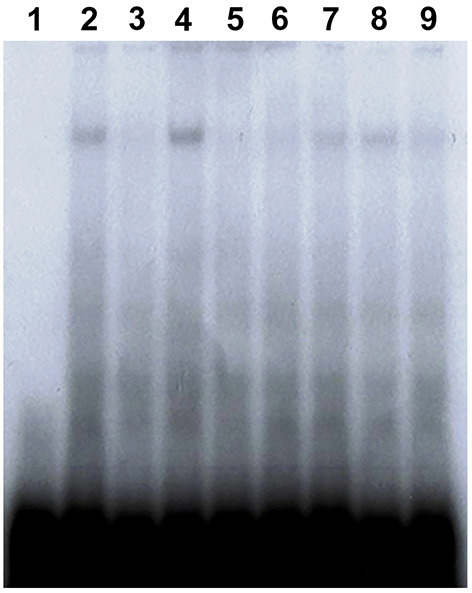
*Vg2*RE-DR2 cis-element compete EcRE and JHRE. EMSAs were performed with *Vg2*RE as a ^32^P-labeled probe (lane 2) in the presence of a 20-fold excess of unlabeled self-competitor (lane 3), EcREs of *hsp*DR4, and *hsp*IR (lanes 5 and 6, respectively) or JHREs of *jhbp21, JHE*, and *CF1/USP* (lanes 7, 8, and 9, respectively). The negative and positive controls were the probe alone without or with nuclear protein (lanes 1 and 4, respectively).

For identification of *PaVg2*RE, a competition assay was performed using 31-bp double-stranded oligonucleotides based on the REs of *hspDR4, hspIR, jhbp21, JHRE, CF1/USP*, and *Vg1*RE (Elgendy et al., 2019) (Supplementary Table 1). The binding of *Vg2*RE by the fat body NPE was effectively abolished by unlabeled *Vg1*RE cold probe and EcREs, DR4, and IR, of *D. melanogaster* (Figure 5, lanes 3, 5, and 6). *Vg2*RE-protein complex was competed by *jhbp21* of *L. migratoria, JHRE*, and *CF1/USP* of *G. mellonella* (Figure 5, lanes 7, 8, and 9, respectively). The JHREs *jhbp21* (*L. migratoria*) and *JHRE* (*C. fumiferana*) showed the lowest competition (Figure 5, lanes 7 and 8).

**Figure 6:**
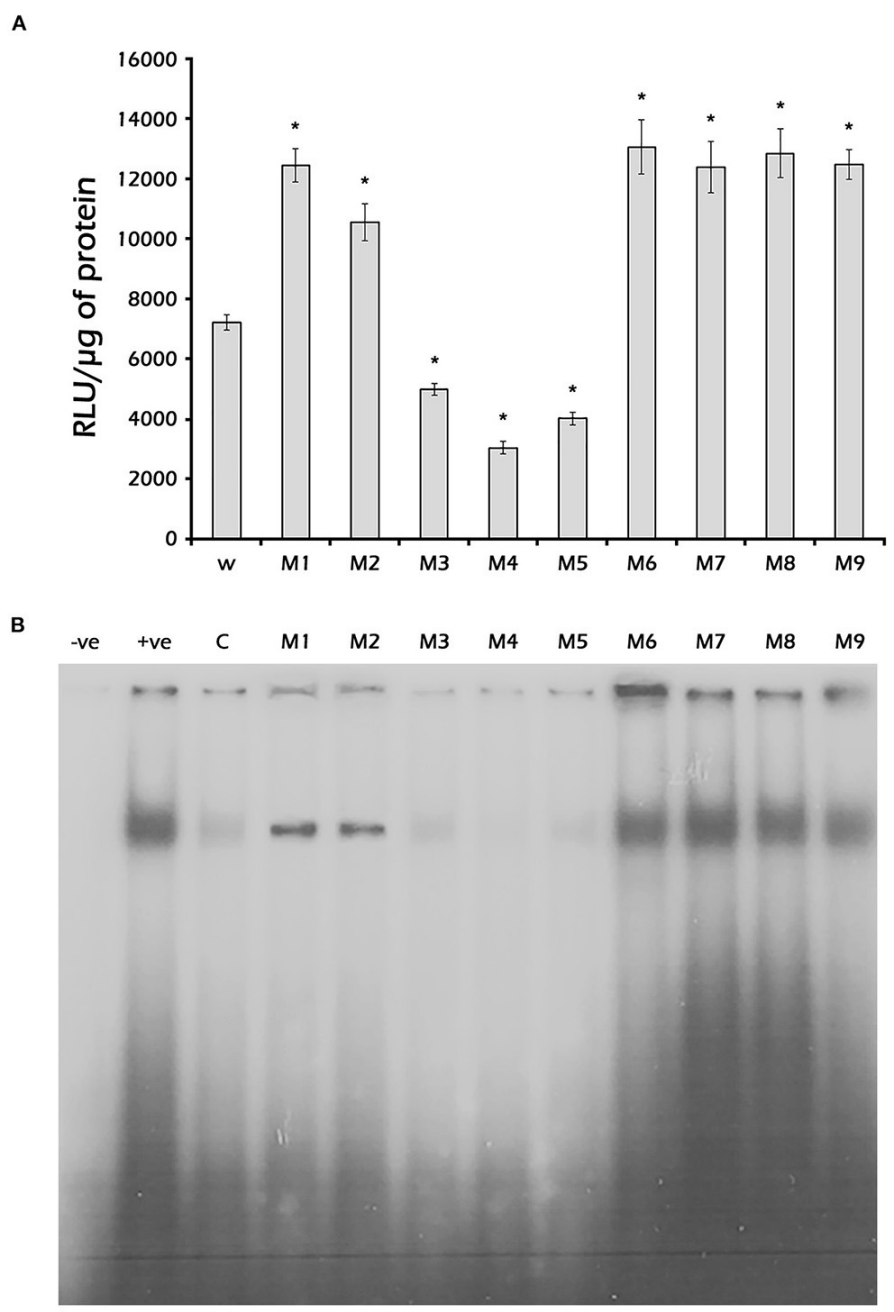
Identification of critical nucleotides base influences the binding of nuclear proteins. **(A)** Luciferase activity: *Sf*9 cells were transfected with the pGL3 −204 bp construct or its mutants (M1–M9) (Supplementary Table 1). Then, the transfected cells were grown in a medium containing 1 μM JH III for 48 h. The cells were harvested, and luciferase activity was measured and normalized to mg protein. Mean ± SD (*n* = 3). **(B)** EMSA: Nuclear proteins were isolated from previtellogenic female fat body cells and incubated with ^32^P-labeled double-stranded 31-bp oligonucleotides probes corresponding to the *Vg2*RE sequence or its mutants (M1–M9) (Supplementary Table 1). The DNA-protein complexes were separated on a 5% non-denaturing polyacrylamide gel. The wild-type 31-bp *Vg2*RE competed with a 50-fold excess of cold probe (lane C) or cold mutants (lanes M1–M9). The negative and positive controls were the labeled wild-type probe (W) without or with nuclear protein (lanes –ve and +ve, respectively). ^*^Statistically significant at *P* < 0.05.

To identify the nucleotides in the *PaVg2*RE (−169 to −139 bp) critical for binding to the fat body nuclear protein (s), the wild-type *Vg2*RE probe used for EMSA was competed with 50-fold excess cold mutant probes (M1–M9) (Supplementary Table 2). The results presented in Figure 6B showed that mutants M3, M4, and M5 competed well with the wild-type (W) probe, indicating that the binding affinity of the probes for the nuclear protein was greater than that of the wild one. M1 and M2 were less competitive than the W, while M6–M9, in which DR2 half-sites or IR were eliminated, showed no competition. This finding indicated that these mutants had no binding affinity for the nuclear protein. SLIM was used to clone different mutated −204 bp constructs (M1–M9) (Supplementary Table 2). Subsequently, the mutated constructs M1–M9 were used to express the firefly luciferase gene in the *Sf*9 cell line in the presence of JH III. The luciferase assay results showed that mutants M3 (G^−166^ and G^−158^ changed to T), M4 (T^−165^ and T^−157^ changed to C), and M5 (G^−168^, G^−169^, G^−161^, and G^−160^ were deleted) reduced the JH III-mediated induction of the reporter gene by 65% compared with the W (Figure 6A). On the other hand, M6–M9 increased reporter gene induction by JH III. These results indicated that the G and T residues of both halves of the *Vg2*RE, DR2 (GAGTCA), and the IR are crucial for binding of the nuclear protein. Furthermore, the luciferase signal decreased as the binding to DR2 increased (Figures 6A,B), which suggests that the expression of *Vg2* is through a binding repression factor/protein.

### Candidate *Vg2*RE Binding Protein (Pull-Down Assay)

**Figure 7:**
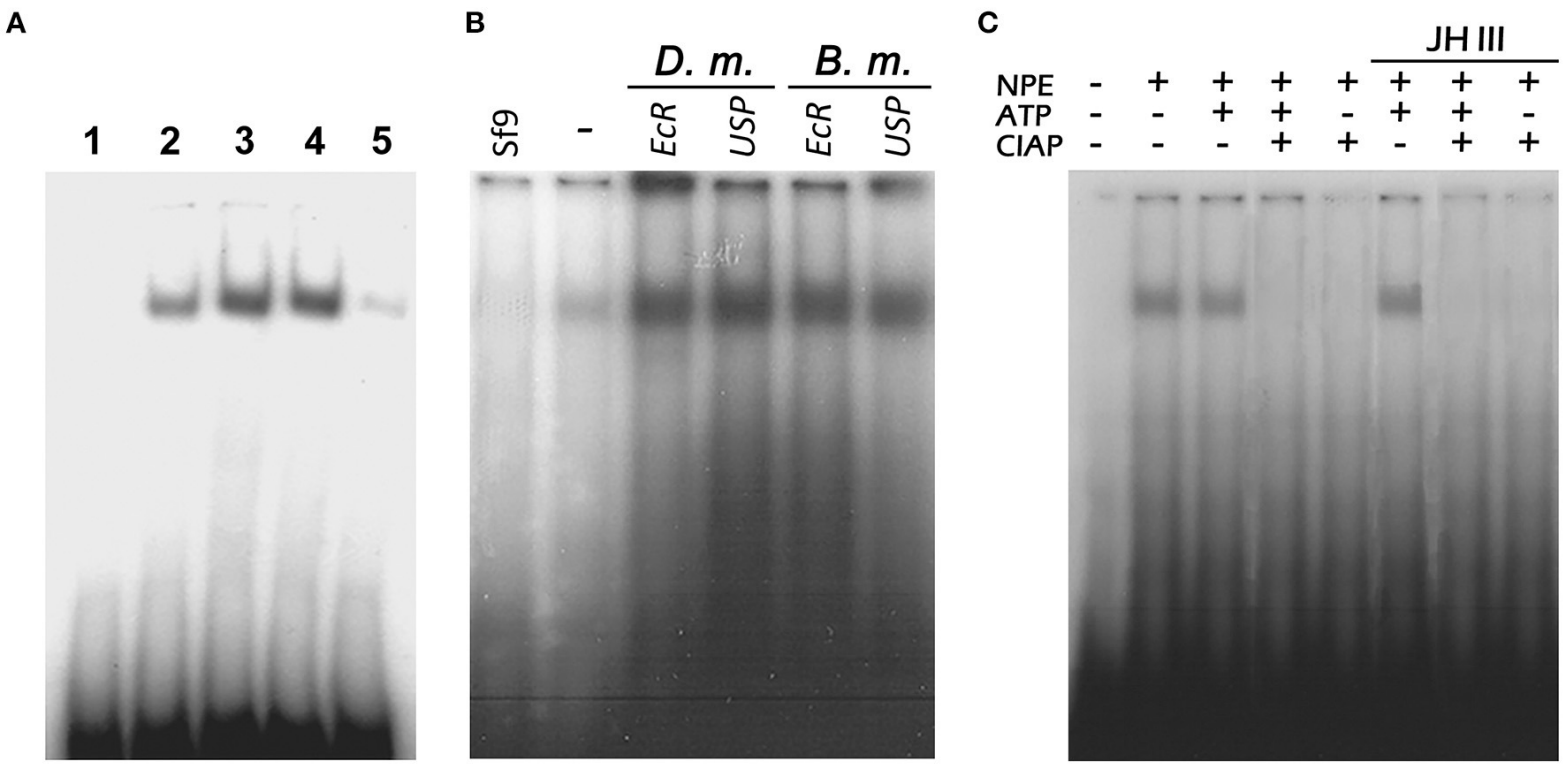
Nuclear proteins binding specifically to *P. americana Vg2*RE (*Vg2*RE). **(A)** Nuclear extracts of the fat body were incubated with ^32^P-labeled 31-bp double-stranded oligonucleotides harboring the *Vg2*RE. The DNA-protein complexes were separated on 5% polyacrylamide gels. Nuclear extracts from nymphs (lane 2) and males (lane 3) showed the same binding complex as previtellogenic female insects (lane 4), while no binding was observed in vitellogenic female insects (lane 5). Lane 1: No nuclear protein extract (NPE). **(B)** NPE from *Sf*9 (lane *Sf*9) and previtellogenic female fat body were incubated with radio-labeled 31-bp double-stranded oligonucleotides harboring the *Vg*2RE in the absence (lane –) or presence of antibodies generated against *D. melanogaster* and *B. mori* EcR and USP before separating on 5% polyacrylamide gel. No super-shifted products were detected. **(C)** Dephosphorylation of the nuclear protein influence binding to the *Vg2*RE. Female fat body nuclear extracts were incubated with either 1 mM ATP, 2 U of CIAP, or 1 mM ATP followed by 2 U CIAP at 22°C for 20 min prior to the binding reaction with the ^32^P-labeled probe containing the Vg2RE. ATP treated NPEs in the presence or absence of JH III showed a binding band when incubated with a *Vg2*RE labeled probe.

An EMSA clearly showed that the NPEs from nymphs, male, and 1-day-old female adults contained proteins that bind specifically to the 31-bp probe (Figure 7A, lanes 2, 3, and 4). The intensity of the *Vg2*RE-NPE complex decreased in the fat body of 5-day old vitellogenic female insects (Figure 7A, lane 5). At this time, there is less nuclear binding protein in the fat body due to the low concentration of JH III in vitellogenic female insects. So, several anti-EcR and anti-USP (the holometabolous *D. melanogaster* and *B. mori*) antibodies were used to characterize the *PaVg2*RE binding protein but no supershifted bands were detected (Figure 7B).

The nuclear protein(s) binds to the radio-labeled *Vg2*RE probe, after incubation with ATP but not after incubation with CIAP (Figure 7C). The addition of JH III to the dephosphorylated NPE cannot restore their binding to the *Vg2*RE probe. This indicated that the presence of JH III cannot directly interfere with the phosphorylation state of NPE (Figure 7C).

**Figure 8:**
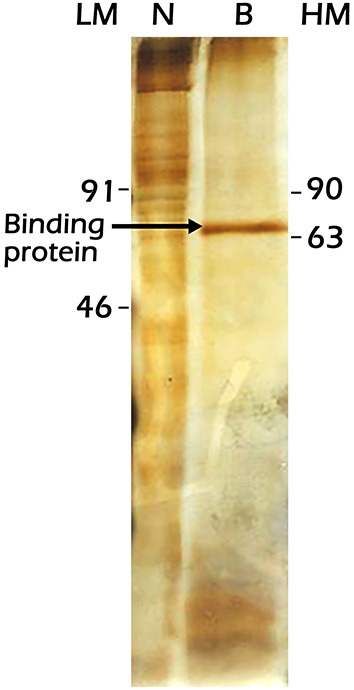
Sodium dodecyl sulfate-polyacrylamide gel electrophoresis (SDS-PAGE) separation and silver staining of the protein precipitated from the previtellogenic female fat body nuclear extracts by the 31-bp *Vg2*RE wild-type sequence. Lane LM: Low molecular weight protein markers, lane N: total nuclear extracts, lane B: protein pulled down by the 31-bp *Vg2*RE, and lane HM: High-molecular-weight protein markers.

In the study, we attempted to identify the *Vg2*RE-binding protein from female fat body nuclear extracts by employing the 31-bp probe (−169 to −139 bp) affixed to the magnetic beads. A 71 kDa protein was isolated as visualized by electrophoretic separation and silver staining (Figure 8). This protein is present in vitellogenic female fat body nuclear extracts but at low concentrations. The purification protocol yielded a 71 kDa protein band in two independent experiments, and in the second experiment, the protein concentration was increased for visualization by the silver staining. This purification was justified as a potential strategy for the isolation and characterization of candidate receptors in upscaled use of materials in the future.

## Discussion

Cockroaches are an ideal model for analyzing the JH molecular signal pathways as it controls gonadotrophic events in adult female and anti-metamorphic events during immature stages (Comas et al., 1999, 2001; Cruz et al., 2003; Maestro et al., 2010; Marchal et al., 2014; Li et al., 2018; Zhu et al., 2020). In adult cockroach species (*B. germanica, D. punctata*, and *P. americana*), JH is known to promote oocyte growth, Vg production in the fat body, and uptake of Vg by the oocytes (Engelmann and Mala, 2000, 2005; Comas et al., 2001). For several decades, the *Vg* gene has been extensively studied due to its variable copy numbers and multiple functions in several oviparous species. *P. americana* has two *Vg* genes with the relative expression level of *Vg2* being more abundant during the female vitellogenic cycle (Tufail et al., 2001). This finding prompted us to inspect the mechanism controlling *Vg* genes expression at the molecular level. To achieve this aim, a genomic fragment of 1,804 bp, 5-flanking region of *Vg2* was cloned, sequenced, and putative binding elements for transcription factors were predicted *in silico* using TRANSFAC database, which contains experimentally validated regulatory elements (AliBaba 2.1, Match and Patch, http://www.gene-regulation.com/pub/programs.html). Others were identified through matching with the known elements (Figure 1; Table 1). Then, luciferase assays and electrophoretic mobility shift, competition, and mutation assays were employed to detangle molecular associations of JH, JH receptors, and coactivators/co-inhibitors and *cis*-elements.

### Transcription Binding Elements in *Vg2* Promoter Region

Multiple putative *cis*-elements for binding transcription factors were detected in the *Vg2* promoter, such as TATA box, JHRE, EcRE, CF-II, FoxO, Hairy, Br-CZs, Met, E74A, E75, E-Box, C-Box, CRE, and tissue-specific element (TSE) (Figure 1; Table 1).

*Periplaneta americana Vitellogenin 2* promoter contains four putative GATA, many GAGA and TSE binding elements which influence the fat body tissue-specific expression of the *Vg2* gene. Similarly, several studies have indicated that the transcriptional activation of *Vg* is regulated by GATA and GAGA factors in *A. aegypti* and *D. melanogaster* (Fossett et al., 2001; Martín et al., 2001; Waltzer et al., 2002; Park et al., 2006). At the regulatory level, the occurrence of E-box motifs and CREs in a promoter region may drive the rhythmic expression of a particular gene(s) (Rund et al., 2013), e.g., aralkylamine *N*-acetyltransferase acting in different aspects of vitellogenesis and the regulation of circadian rhythms of locomotor activity in *P*. *americana* (Kamruzzaman et al., 2020, 2021). CRE-dependent transcription and cAMP signaling constitute integral components of core molecular clocks, serving to regulate daily rhythmic transcription of circadian clock genes/clock-controlled genes and signal transduction of biogenic amines and neuropeptides (Belvin et al., 1999; Vleugels et al., 2013; Fernandez-Chiappe et al., 2020). In *A. aegypti*, a conserved E-box motif, contained within JHRE, is required for JH induction of Kr-h1 (Cui et al., 2014).

Many putative binding elements for ecdysone and ecdysone cascade genes such as Br-CZs, E74A, and E75 were also identified in the *PaVg2* promoter. Both Br-CZ and E74A are involved in many important processes in insects, such as metamorphosis, oogenesis, and vitellogenesis (Chen et al., 2004; Dubrovsky, 2005; Spokony and Restifo, 2007). Br-CZ is involved in the reproductive regulation in the mosquito *A. aegypti, B. mori, Bombus lantschouesis*, and the cockroach *B. germanica* (Piulachs et al., 2010; Cruz et al., 2012; Hansen et al., 2014; Yang et al., 2014; Lin et al., 2017), and its functions are closely related to the ecdysone signaling pathway.

Also, the *PaVg2* promoter region contains four putative binding elements for JH and its candidate receptor Met (one element) and Hairy (four elements). There were multiple putative binding elements for transcription factors, FoxO and CF-II, which might act as repressors or enhancers, according to their phosphorylation state (Sok et al., 2008; Sheng et al., 2011; Wu et al., 2020). FoxO silencing in *B. germanica*, resulted in increased Vg mRNA levels in the fat body and a rise in JH biosynthetic activity (Suren-Castillo et al., 2012) and. FoxO also enhanced polyploidy of fat body cells of *L. migratoria* in preparation for the large-scale Vg synthesis required for synchronous maturation of multiple eggs (Wu et al., 2020). FoxO silencing in *B. germanica*, resulted in increased Vg mRNA levels in the fat body and a rise in the JH biosynthetic activity (Suren-Castillo et al., 2012). FoxO also enhanced polyploidy of fat body cells of *L. migratoria* in preparation for the large-scale Vg synthesis required for synchronous maturation of multiple eggs (Wu et al., 2020).

### Detection of DR2 Element in the Proximal *Vg2* Promoter Region

Based on the luciferase reporter experiments, the promoter region of *PaVg2* can be divided into the proximal region (−1 to −204 bp) with many putative binding elements which activate or induce the *PaVg2* expression and the second middle region (−204 to −1,548 bp) showing an intermediate induction of the luciferase assay due to presence of putative binding elements for both JH and 20E and their early and late response proteins hierarchy. Finally, the distal region (−1,548 to −1,804 bp) showed the highest induction level (Figure 3).

The luciferase assay of the −1,404 bp construct showed a significant reduction almost equal to the control construct even in the presence of JH III. EMSA using this 145-bp inhibitory probe (from −1,404 to −1,240 bp) showed no binding with NPE of the vitellogenic fat body of the female insects (data not shown). This finding suggests that 20E represses the cockroach *Vg2* gene *via* 20E-induced TF protein response elements present in the fragment.

Detailed inspection for the −204 bp constructs that gave the highest luciferase activity revealed a 6 bp direct repeat with 2-nucleotide spacer DR2, GAGTCA (^−168^GAGTCA CGGAGTCG^−155^), which overlapped with 10 bp inverted repeat, IR (^−158^GTCGCCGCTG^–149^). This identified DR2 is homologous to many of holometabolous hormone response elements: direct repeat half-site of DR4 (AGGTCA) from the *D. melanogaster* heat shock protein 27 gene (*hsp27*), JH binding protein (*jhbp*) response element (AGGGTCACTACCATA) of *G. mellonella* JH binding protein gene, juvenile hormone esterase response element (JHERE) (AGATTATTATAGATTA) of *C. fumiferana* JH esterase gene and juvenile hormone-binding protein 21 (*jhbp21*) *(*GAGGTTCGAG^A^/_T_CCT^T^/_C_) of *L. migratoria JH binding protein 21 gene*. These DR2s are 95% homologous to *Vg1* DR2 identified from *P. americana* (Elgendy et al., 2019).

The mutants of the *Vg2*RE 31-bp probe were used to conduct luciferase and competition EMSA assays. The results showed that mutants with substitutions of the first G to A and T to C in both half-sites of the DR2 GAGTCA competed well with the wild-type probe, which subsequently resulted in the reduction of reporter activity. Other mutants and deletion of any half-site of DR2 and IR did not show any competition with the wild-type probe. These results indicate that the G and T bases are the most important residues. Kethidi et al. (2004) showed that oligonucleotides containing two AGGTCA elements that are separated by a 4-nt spacer competed well with wild probe (AGATTATTATAGATTA).

In the EMSA, the *Vg2*RE-binding complex was effectively completed away by EcRE DR4 and IR of *D. melanogaster* and JHRE CF1/USP of the *G. mellonella jhbp* gene cold probes. Moreover, the binding complex was eliminated by *Vg1*RE of *P. americana*. These results may explain why this binding complex plays a role in the JH and 20E hormonal crosstalk. Because the identified *Vg2*RE contains a conserved DR2 and IR response element, the proteins that bind to these elements likely belong to the nuclear receptor superfamily (Kethidi et al., 2004), such as EcR and Ultraspiracle (USP). However, the NPE–*Vg2*RE complex did not show any supershift results using *D. melanogaster* and *B. mori* EcR and USP antibodies. This result may be due to low homology between holometabolous nuclear receptor EcR and USP and those of cockroaches.

### Inductive/Repressive Roles of *Vg2*RE-DR2 Element

Electrophoretic mobility shift assay results showed that NPE of nymph, male, and previtellogenic female fat bodies bound specifically to the *Vg2*RE probe but that of vitellogenic female NPEs did not (Weaver et al., 1984; Weaver and Edwards, 1990). We, therefore, postulate that binding of a candidate nuclear protein to *Vg*2RE leads to the repression of *Vg2* expression in nymph and male *P. americana*. However, this needs detailed study in the future. Similarly, Sheng et al. (2011) found that binding of FoxO to *T. castaneum Vg2* gene promoter inhibited its transcription but after adult beetle emergence, JH stimulated FoxO phosphorylation, which in turn released FoxO binding and induced *Vg2* transcription. Other binding repression has been detected for the CF1/USP element II found in the *jhbp* gene (resembling the 5′ half-site of EcRE) showing an inhibitory effect on the transcriptional activity of the *jhbp* promoter. Thus, it may be concluded that CF1/USP elements play a repressive function in the *jhbp* gene (Sok et al., 2008). Also, in the differentiation of the imaginal tissues, the unliganded ecdysone receptor acts as a repressor to interrupt the sequence of differentiation at different points, coordinating the response to the rising 20E titers. The release of repression by 20E may, therefore, function as a gate at the onset of metamorphosis (Schubiger et al., 2005). In *D. punctata* knock down of the JH receptor, *Met* at the first gonadotropic cycle effectively reduced the expression of Vg in the fat body and oocysts maturation (Marchal et al., 2014).

### A Putative NPE Binding to *Vg2*RE-DR2

Electrophoretic separation and silver staining visualization of the bound nuclear protein pulled down by the 31-bp *Vg2*RE probe showed a 71 kDa protein. This protein exhibits specific binding and could be a partner of the hormone-receptor complex. *Vg2*RE contains a conserved DR2 and IR response element; the proteins that bind to this JHRE likely belong to the nuclear receptor superfamily (Kethidi et al., 2004).

Zhu et al. (2020) postulated that double sex protein, Dsx, might be a molecular link in the JH regulation of vitellogenesis in *P. americana*. However, Dsx-RNAi did not completely abolish JH-induced *Vg* expression in the *P. americana* fat body suggesting that other transcriptional factors, i.e., GATA (Park et al., 2006) could be involved in the JH induction of *Vg* expression.

One of the candidate binding proteins of *Vg2*RE (DR2) is CF1/USP protein, because in the competition EMSA, the *Vg2*RE-NPE complex was effectively abolished by unlabeled EcREs DR4 and IR of *D. melanogaster* and JHREs CF1/USP of *G. mellonella, jhbp*21 (Figure 7B). An interaction between USP and DNA relies on recognition of the consensus sequence (GGGTCA) and the ionic interactions of several phosphate groups outside from this element (Sok et al., 2008; Niewiadomska-Cimicka et al., 2011). The results in Figure 7C suggest that only the phosphorylated nuclear protein binds to *Vg*2RE(DR2) and the addition of JH III does not interfere the phosphorylation state of the NPE (Figure 7C). This suggests another direct or indirect role of JH III at the cellular level other than the nucleus. In *T. castaneum Vg2* expression is induced after FoxO phosphorylation which is stimulated by JH (Sheng et al., 2011). Other nuclear binding protein candidates are Met or Taiman, which suppressed Vg gene expression of the linden bug, *P. apterus* when one or both are knocked down (Smykal et al., 2014). The different JH binding protein(s) suggested that JH has a common receptor but different partners in the transduction of JH signals. JH also can act *via* plasma membrane receptors (Davey, 2000; Liu et al., 2015). In *D*. *melanogaster*, JH induces protein expression in the accessory glands by Met, PKC, and calcium (Yamamoto et al., 1988; Wilson et al., 2003). It will be important to understand how both the intracellular and membrane receptors of JH interact to regulate metamorphosis and reproduction in insects.

## Conclusions

We cloned the *Vg2* promoter from the American cockroach as a model of a JH-dependent gene. Then, we identified hormone response element homologs of the DR4 of the *D. melanogaster* EcR response element using both the luciferase reporter assay and EMSA. Additionally, we detected a 71 kDa protein that binds to this identified response element. The EcR response elements and *Vg2*RE share the half-sites AGGTCA, which indicates that these elements also share a transcriptional regulatory protein that plays a role in both JH and ecdysone hormone signaling in insects, depending on the target tissue and the developmental stage. The results represent the first attempt to describe functional elements in the *Vg* gene from a hemimetabolous insect and provide a necessary step for elucidation of the complexity of JH-induced gene expression. As it is possible that different cell lines or hormone treatments could yield different results, it would be of great interest to clone many of the candidate transcription factor protein genes for use in RNA interference experiments to elucidate the hormone signal transduction mechanisms of JH.

## Data Availability Statement

The original contributions presented in the study are included in the article/Supplementary Material, further inquiries can be directed to the corresponding author/s.

## Author Contributions

MaT and AE designed the research. AE, AM, and BD performed different aspects of the formal analysis and wrote the original draft. MaT, BD, and AM reviewed and edited the final drafts. All the authors approved the submission of this manuscript.

## Conflict of Interest

The authors declare that the research was conducted in the absence of any commercial or financial relationships that could be construed as a potential conflict of interest.

## Publisher's Note

All claims expressed in this article are solely those of the authors and do not necessarily represent those of their affiliated organizations, or those of the publisher, the editors and the reviewers. Any product that may be evaluated in this article, or claim that may be made by its manufacturer, is not guaranteed or endorsed by the publisher.

## Supplementary Material

Supplementary Table 1 List of primers and probes used in the present study.

Supplementary Table 2 The wild-type (*Vg2*W) and mutant sequences (M1–M9) used in this study.
