# Supplementary material for: Involvement of Cis-Acting Elements in Molecular Regulation of JH-Mediated Vitellogenin Gene 2 of Female Periplaneta americana
Source: Front Physiol. 2021 Aug 30;12:723072. doi: 10.3389/fphys.2021.723072 (PMC8435907; doi:10.3389/fphys.2021.723072)
Supplement: Supplementary Table 1 — List of primers and probes used in the present study. [file Table_1.DOCX]

**Supplementary Table 1.** List of primers and probes used in the present study

| Experiment | Primer Name | Sequence |
| --- | --- | --- |
| Promoter sequence | *Vg2*-Prom1  *Vg2*-Prom2 | GGACACAGCCAGCAGACAGCAGAGG (+18 to +38)  CAGAGGAGCGTCTTCCACATCATCTT (+33 to +57) |
| Primer extension analysis | *Vg2*p-R | GCAGAGGAGCGTCTTCCACATC (+18 to +39)  (*Vg2* cDNA sequence, Tufail et al., 2001) |
| Reporter constructs | –1804 bp Con  –1548 bp Con  –1404 bp Con  –1237 bp Con  –870 bp Con  –347 bp Con  –204 bp Con  –177 bp Con  –139 bp Con  –74 bp Con | GAGCTC**TTGGAGCGAGTGTTATTGTCTG**  GAGCTC**GCCTGTCTAAATTTCGTCCCC**  GAGCTCC**CGTAAAACCTGTTAGGGAGA**  GAGCTC**AGGCGTGGTACAGCAGTTCTA**  GAGCTC**TTGTGACGTGTCTGAATGAGTG**  GAGCTC**ATATTTGAGTAATATCCAACCG**  GAGCTC**GCTGAGTTGTCCATATAAAACA**  GAGCTC**CGTAGAAGGGAGTCACGGAGGTC**  GAGCTC**TGCTCTGCATTATTTTGACAT**  GAGCTC**CCAGTCTCCATTCGACCCGCT** |
| Basic pGL3 | RV3 primer  GL 2 primer | CTAGCAAAATAGGCTGTCCC  CTTTATGTTTTTGGCGTCTTCCA |
| Probes for EMSA | *Vg1*RE  *Vg2*RE  hspDR4  hspIR  jhbp21  JHRE  CF1/USP | TGCGACCTCGTGACCTCGTGCACCAAGGGCT  AGAAGGGAGTCACGGA*GTCGCCGCTG*GTTTC  CTTATCAGAGGTTCGAGACCTCCCTCAGGC  GCGACAAGGGTTCAATGCACTTGTCCATTG  AGAGACAAGAGGTCAATGACCTTGTCCAA  CCCTTATAAAAAGATTATTATAGATTATTA  AGTTACAGGGGGTCAAAAGTACCTACTGAG |

The SacI site is underlined in the sense primer sequences.

Inverted repeat is italic and dashed underlined.
