# Supplementary material for: Involvement of Cis-Acting Elements in Molecular Regulation of JH-Mediated Vitellogenin Gene 2 of Female Periplaneta americana
Source: Front Physiol. 2021 Aug 30;12:723072. doi: 10.3389/fphys.2021.723072 (PMC8435907; doi:10.3389/fphys.2021.723072)
Supplement: Supplementary Table 2 — The wild-type (Vg2W) and mutant sequences (M1–M9) used in this study. [file Table_2.DOCX]

**Supplementary Table 2**: The wild-type (*Vg2*W) and mutant sequences (M1-M9) used in this study

| Name | Sequence |  |
| --- | --- | --- |
| Vg2W | AGAAGGGAGTCACGGAGTCGCCGCTGGTTTC |  |
| M1 | AGAAAGAAGTCACGGAGTCGCCGCTGGTTTC |  |
| M2 | AGACGGGCGTCACGGAGTCGCCGCTGGTTTC |  |
| M3 | AGAAGGGATTCACGGATTCGCCGCTGGTTTC |  |
| M4 | AGAAGGGAGCCACGGAGCCGCCGCTGGTTTC |  |
| M5 | AGAAGggAGTCACggAGTCGCCGCTGGTTTCGATG |  |
| M6 | AGAAGGgagtcaCGGAGTCGCCGCTGGTTTCGATGTG | (Del 1^st^ half) |
| M7 | AGAAGGGAGTCACGgagtcgCCGCTGGTTTCGATGTG | (Del 2^nd^ half) |
| M8 | AGAAGGGAGTCACGGAGTCGCCGCTAGTTTC | (IR) |
| M9 | AGAAGGTAATCACGTAATCGCCGCTGGTTTC |  |

*Vg2*RE DR2 is underlined.

Mutated nucleotides indicated by grey boxes.

Deleted nucleotides in small letters.
